# Right Ventricular Compression Mimicking Brugada-Like Electrocardiogram in a Patient with Recurrent Pectus Excavatum

**DOI:** 10.1155/2017/3047937

**Published:** 2017-02-20

**Authors:** Jinhee Ahn, Jong-Il Choi, Jaemin Shim, Sung Ho Lee, Young-Hoon Kim

**Affiliations:** ^1^Division of Cardiology, Department of Internal Medicine, Korea University College of Medicine and Korea University Medical Center, Seoul, Republic of Korea; ^2^Division of Cardiology, Department of Internal Medicine, Pusan National University Hospital, Busan, Republic of Korea; ^3^Department of Thoracic and Cardiovascular Surgery, Korea University Medical Center, Seoul, Republic of Korea

## Abstract

Pectus excavatum (PE), the most common skeletal anomaly of chest wall, sometimes requires a surgical correction but recurrent PE is not uncommon. PE usually has a benign course; however, this chest deformity may be associated with symptomatic tachyarrhythmias due to mechanical compression. We report a case of a patient with recurrent PE after surgical correction presenting with palpitation and electrocardiogram (ECG) showing ST-segment elevation on the right precordial leads, which could be mistaken for a Brugada syndrome (BrS).

## 1. Introduction

Pectus excavatum (PE) is the most common congenital deformity of the anterior chest wall with male predominance [1]. Surgical correction with modified Ravitch or Nuss technique is sometimes performed, but recurrent PE is not uncommon [1]. Indications of the operation for PE are various, of those, symptomatic tachyarrhythmia is one of the important reasons [2]. As a consequence of mechanical compression, PE leads to structural or hemodynamic consequences which may be proarrhythmic [2, 3]. We report a case of young man with recurrent PE after the first surgical correction at the age of 3. He presented with palpitation and his electrocardiogram (ECG) showed ST-segment elevation on the right precordial leads, which could be mistaken for a Brugada syndrome (BrS).

## 2. Case Report

**Figure 1:**
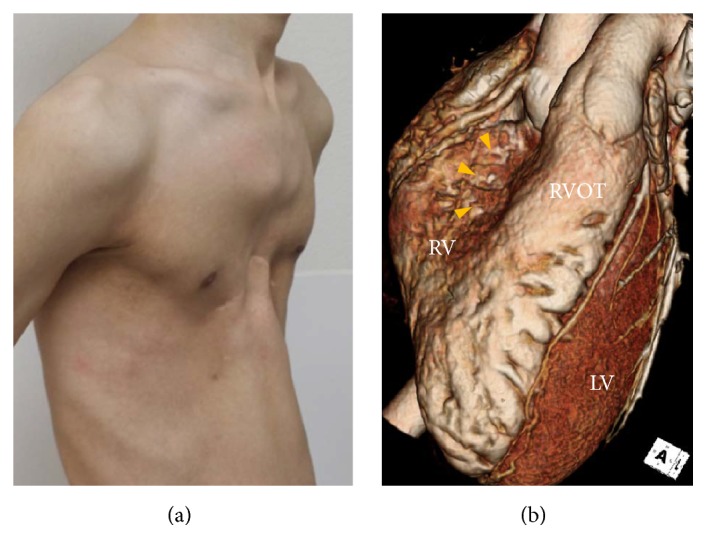
(a) Gross picture shows a deformity of the anterior thoracic cage consistent with pectus excavatum. (b) 3D reconstructed computed tomography image also reveals external compression of basal-to-mid portion of the right ventricle (yellow arrow heads). LV, left ventricle; RV, right ventricle; RVOT, right ventricular outflow tract.

**Figure 2:**
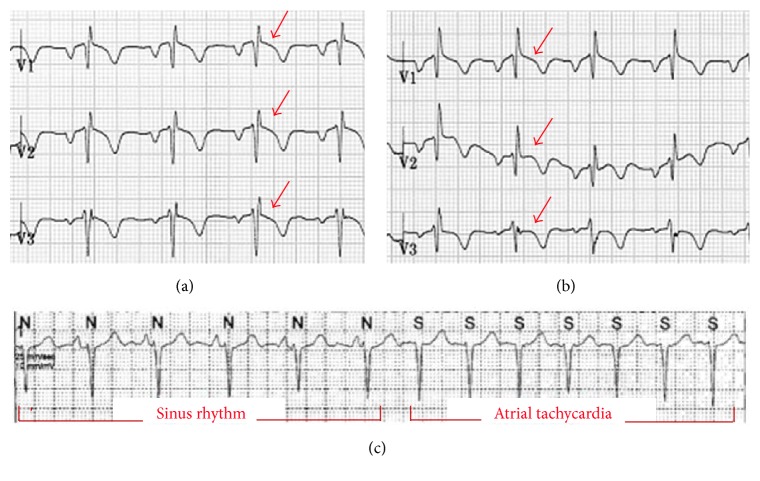
(a) Electrocardiogram (ECG) shows an rSR' pattern with ST-segment elevation (red arrows) and T-wave inversion on the right precordial leads, indicating a Brugada-like ECG pattern. (b) Six months after Nuss operation even though ST-segment elevation is not fully recovered on lead V_1_, it seems to be normalized on lead V_2-3_. (c) Atrial tachycardia was documented on twenty-four-hour ambulatory Holter monitoring before the operation.

**Figure 3:**
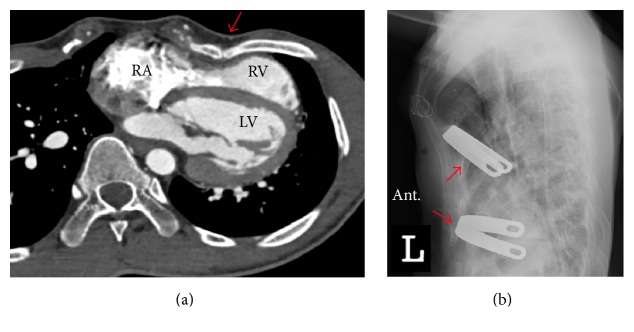
(a) Cross-sectional computed tomography image shows external compression (red arrow) on the basal-to-mid portion of the right ventricle (RV) and dilation of the right atrium and RV apical site (Haller index 5.21). (b) A lateral view of the chest X-ray after Nuss operation shows that anterior chest wall is decompressed by two Nuss bars underneath the sternum (red arrows) across the chest. RA, right atrium; RV, right ventricle; LV, left ventricle; ant., anterior.

A 23-year-old man presented with palpitation, which occurred abruptly during resting state and lasted 10 minutes. He has been healthy except a past history of surgical correction for PE 20 years prior. He denied having episodes of syncope or a family history of sudden cardiac arrest. At present, physical examination revealed a deformity of the anterior thoracic cage consistent with PE (Figure 1(a)). ECG revealed an rSR' pattern with slight ST-segment elevation and T-wave inversion on the right precordial leads, indicating a Brugada-like ECG pattern (Figure 2(a)). Laboratory findings were within normal range. Both signal-averaged ECG and flecainide challenge test showed no significant findings. During treadmill testing, no tachyarrhythmia was noted except an infrequent single ventricular premature beat at stage 4. Twenty-four hour ambulatory Holter monitoring revealed several episodes of sustained atrial tachycardia with a maximum duration of 5 minutes (Figure 2(c)). Echocardiography and cardiac computed tomography demonstrated external compression on the basal-to-mid portion of the right ventricle (RV) and dilation of the right atrium and RV apical site with mild hypokinesia (Figures 1(b) and 3(a)). Haller index which is defined as the ratio of the transverse (the horizontal distance of ribcage) and the anteroposterior diameter (the shortest distance between the sternum and vertebrae) was 5.21. Based on these data, the patient's symptom was most likely due to atrial tachycardia. Nuss operation was carried out, and postoperative chest X-ray demonstrated effective prevention of sternal compression (Figure 3(b)). Follow-up echocardiography revealed that the RV was decompressed, leading to normal contractility. During a six-month follow-up period, no symptoms were reported and no tachyarrhythmias were documented. ST-segment morphology seemed to be normalized on lead V_2-3_ (Figure 2(b)).

## 3. Discussion

To the best of our knowledge, this is the first case of PE provoking atrial tachycardia and mimicking a Brugada-like ECG pattern via extrinsic compression of the RV, even though he underwent a prior operation for chest wall deformity. The first surgical correction performed at the age of 3 could not keep pace with chest wall expansion as the patient matured.

Indications of surgical correction for PE included cosmetic matter, progression of the deformity, Haller index > 3.0, structural compression or displacement resulting in paradoxical movement of the chest wall or any cardiac pathology, or frequent symptoms such as dyspnea, palpitation, or easy fatigue [4]. However, recurrent PE is not uncommon. One of the reasons for recurrence is surgical timing. Usually repair is postponed until the later stages of teenage to allow complete skeletal maturity [5]. Younger children with cardiopulmonary compromise may be candidates for early repair as our patient. Nevertheless, deformity correction at too early of the age can lead to improper growth of chest wall and then have a higher chance of recurrence [6]. Other suggested reasons of PE recurrence include too extensive or too small dissection, inherent cartilage dysfunction, ossified costal cartilages, or tight adhesion between chest wall soft tissue and bone after operation [1].

PE presents with various ECG abnormalities including symptomatic tachyarrhythmias such as ventricular tachycardia that rarely require catheter ablation [4]. In this patient atrial tachycardia was documented. Probably it was provoked due to atrial bulging and hemodynamic changes caused by chronic mechanical compression on RV. Because this compression was considered as a source of arrhythmogenesis, surgical correction was performed and then no more tachycardia has been documented thereafter.

Brugada phenocopy (BrP), a recently established clinical phenomenon that is distinct from BrS, can be caused by mechanical mediastinal compression on RV [7]. BrP is defined as a typical Brugada morphology in the absence of true BrS and this pattern resolves once the underlying predisposing condition is relieved [8]. In this patient, BrS was suspected based on well-defined peaked *r*′, slight ST-segment elevation combined with T-wave inversion on the right precordial leads. In addition, the ratio of amplitude of ST-segment at J-point and the point 80 ms after J-point was higher than 1 and QRS duration in left leads was not wider than 120 ms [8]. However, it was less likely in that flecainide provocation testing was definitely negative and there was no history of syncope, aborted cardiac arrest, or family history compatible with BrS. Also, even though ST-segment morphology was not fully recovered yet, it seemed to be normalized after the surgical correction. The mechanism of BrP in PE is suggested to be most likely due to chronic mechanical compression, anatomical displacement, and rotation which affect multiple ion channels leading to increase in outward currents during the early phase of the epicardial action potential [7, 9].

As shown in this case, PE is one of the causes of BrP and it can also provoke atrial tachycardia. Also, before surgical correction for PE, proper operation timing should be deliberate. While PE is typically considered a benign condition, surgical correction may be needed if associated cardiac symptoms appear even after a prior correction [9].
